# Genome Assembly of the Cold-Tolerant Leaf Beetle *Gonioctena quinquepunctata*, an Important Resource for Studying Its Evolution and Reproductive Barriers between Species

**DOI:** 10.1093/gbe/evab134

**Published:** 2021-06-11

**Authors:** Svitlana Lukicheva, Jean-François Flot, Patrick Mardulyn

**Affiliations:** Evolutionary Biology and Ecology & Interuniversity Institute of Bioinformatics in Brussels – (IB)2, Université Libre de Bruxelles (ULB), Brussels, Belgium

**Keywords:** Chrysomelidae, whole-genome sequence, de novo assembly, genome annotation

## Abstract

Coleoptera is the most species-rich insect order, yet is currently underrepresented in genomic databases. An assembly was generated for ca. 1.7 Gb genome of the leaf beetle *Gonioctena quinquepunctata* by first assembling long-sequence reads (Oxford Nanopore; ± 27-fold coverage) and subsequently polishing the resulting assembly with short sequence reads (Illumina; ± 85-fold coverage). The unusually large size (most Coleoptera species are associated with a reported size below 1 Gb) was at least partially attributed to the presence of a large fraction of repeated elements (73.8%). The final assembly was characterized by an N50 length of 432 kb and a BUSCO score of 95.5%. The heterozygosity rate was ± 0.6%. Automated genome annotation informed by RNA-Seq resulted in 40,568 predicted proteins, which is much larger than the typical range 17,000–23,000 predicted for other Coleoptera. However, no evidence of a genome duplication was detected. This new reference genome will contribute to our understanding of genetic variation in the Coleoptera. Among others, it will also allow exploring reproductive barriers between species, investigating introgression in the nuclear genome, and identifying genes involved in resistance to extreme climate conditions.

Significance

Coleoptera, the most species-rich insect order, is currently underrepresented in genomic databases. We generated a de novo genome assembly and genome annotation for a new Coleoptera species, the cold-resistant leaf beetle *Gonioctena quinquepunctata* (Fabricius, 1787). The assembly produced was characterized by a size of 1.7 Gb and the genome was predicted to encode more than 40,000 proteins. These numbers are unusually high for insects, even for chrysomelids (that seem to harbor particularly large genomes), and are at least partially explained by the presence of a large fraction of highly repetitive DNA. This new reference genome will allow important advances in our understanding of evolution and speciation in chrysomelid beetles.

## Introduction

With more than 340,000 described species, the order Coleoptera has by far the highest number of species of all insect orders (Hespenheide 2001; Mayhew 2002). This exceptional species richness has been attributed to various causes, including an adaptive radiation associated with multiple shifts to specialized herbivory on a large diversity of angiosperm species (Farrell 1998); horizontal transfers of plant cell wall-degrading enzymes from bacteria and fungi (McKenna et al. 2019); and an exceptionally low rate of extinction within the clade Polyphaga (Smith and Marcot 2015). Despite this high species richness, the number of beetle species for which a genome assembly is currently available remains markedly lower than for other insect orders such as Hymenoptera or Diptera (Thomas et al. 2020). Here, we present the first genome assembly of *G. quinquepunctata*, a member of Chrysomelidae (which is one of the largest beetle families and encompasses ± 35,000 described species; Hespenheide 2001).

A cold-tolerant insect with a widespread but fragmented distribution across Europe, *G. quinquepunctata* can be used as a model to study the impact of climate variation that occurred at the end of the Pleistocene. Although it is well differentiated from its sister species *Gonioctena intermedia*, both species display parapatric distributions, sharing a portion of their range mainly inside the Alps. It was shown that both species occasionally hybridize where they meet and that as a consequence, introgression of the mitochondrial genome has occurred multiple times from *G. quinquepunctata* to *G. intermedia* (Quinzin and Mardulyn 2014).

Our new assembly, the 15th among beetles and the 5th among chrysomelids, provides an important resource for studying the evolution of the range of this cold-tolerant species in response to past climate changes, and for studying its mechanism of speciation at the genome level. This paves the way for comparing genomic variation within and between *Gonioctena* species, allowing to identify regions of strong differentiation that have potentially played a role in the emergence of reproductive barriers between the two species and to characterize the amount of introgression between them.

## Results and Discussion

### Genome Characteristics Estimation

Prior to assembling the genome of *G. quinquepunctata*, we used k-mer-based approaches to estimate its size. We found it to be ≈1.7 Gb (GenomeScope: 1.56, kmercountexact: 1.9), which is larger than that of most Coleoptera species, reported to be below 1 Gb (Petitpierre et al. 1993; Hanrahan and Johnston 2011). The heterozygosity rate was estimated at ca. 0.6% using both GenomeScope and kmercountexact.

### Genome Assembly and Gene Prediction

Based on the genome size estimate above, the 46 Gb of Nanopore reads and 145 Gb of Illumina paired-end reads we generated from a single individual correspond to, respectively, a 27-fold and an 87-fold coverage of the genome. The percentage of 1,658 single-copy orthologs from the Insecta data set was 53.4% in the raw contigs then 60.7% after the first polishing step and 96% after the second one. The assembly consisted of 24.7 million contigs, with a total length of 1.9 Gb and an N50 length of 359 kb. Running Purge Haplotigs decreased the number of contigs to 10 million and the length of the assembly to 1.7 Gb, whereas its N50 reached 432 kb. Purge Haplotigs also decreased the number of k-mers represented twice in the assembly (supplementary fig. 1, Supplementary Material online), while slightly decreasing the k-mer completeness from 96.25% to 95.69%. The final assembly contained 95.5% complete, 2.2% fragmented, and 2.3% missing orthologs (table 1).

**Table 1:** Summary of Assembly Statistics

Assembly	Size (Mb)	1,732
	Number of contigs	10,033
	Number of contigs >50 k	5,755
	Longest contig (Mb)	3.03
	Contig N50	4,32,124
	*N* (%)	0
	GC (%)	34.61
BUSCO	Complete (%)	95.5
	Complete duplicated (%)	2
	Fragmented (%)	2.2
	Missing (%)	2.3
Repetitive elements	Total (%)	66.09
	SINEs (%)	0
	LINEs (%)	13.76
	LTR (%)	4.9
	DNA transposons (%)	11.98
	Unclassified (%)	42.22
Annotation	Predicted genes	38,493
	Predicted proteins	40,568
	Functionally annotated	19,357
	Mean gene length	15,141
	Mean exon length	267
	Mean intron length	6,479
	Exons per gene	3.53
	Introns per gene	2.53

A total of 73.8% of the assembly was identified as composed of repeated regions, which is higher than the 64% identified for *Callosobruchus maculatus* (Sayadi et al. 2019) and the 58% for *Ophraella communa* (Bouchemousse et al. 2020). A high proportion of the repetitive elements identified in the genomes of the latter two species (54% for *C. maculatus* and 68% for *O. communa*) could not be classified, which the authors of these studies interpreted as possibly reflecting long evolutionary distances to previously known repeats. This value was lower (42%) for *G. quinquepunctata*, but still represents a large amount (table 1).

The annotation pipeline identified 39,463 coding genes and 41,598 proteins. After all proteins with missing start or stop codons were removed, these values decreased slightly to 38,493 and 40,568. We were able to annotate 19,357 (47.7%) of these proteins by reference to the Swiss-Prot and InterPro databases. Among the 31,981 (78.8%) proteins that had strong matches against the NCBI NR database, 26,176 (82%) of them were mapped to beetle proteins (with 13,179 [41%] matches to *Leptinotarsa decemlineata*, 3,458 [11%] to *Anoplophora glabripennis* and 2,618 [8%] to *Diabrotica virgifera virgifera*). Bacteria and virus proteins matched, respectively, 144 and 41 proteins predicted for *G. quinquepunctata* (for a total of 0.6%), suggesting a very low-level bacterial contamination.

The number of predicted proteins (40,568) is much larger than the range 17,000–23,000 predicted for other Coleoptera (Cunningham et al. 2015; Vega et al. 2015; Meyer et al. 2016; Evans et al. 2018; Schoville et al. 2018; Sayadi et al. 2019; Herndon et al. 2020), with the exception of the recently published genome of *O. communa* (Bouchemousse et al. 2020) that was associated with an even higher number of predicted proteins (75,642). The authors of this study considered this unusually high number of predicted proteins as a probable overestimation resulting from the high number of transposable elements found in this genome, many of which were not currently included in the database. Many of these predicted proteins may have therefore been undetected transposons. Because the proportion of repetitive elements is even higher in the genome of *G. quinquepunctata*, a similar hypothesis can be proposed. We investigated the alternate possibility that the genome of *G. quinquepunctata* was actually polyploid, but MCScanX detected only 22 collinear genes, which did not provide any evidence in support of this hypothesis.

### Phylogenetic Analysis

**Fig. 1.**
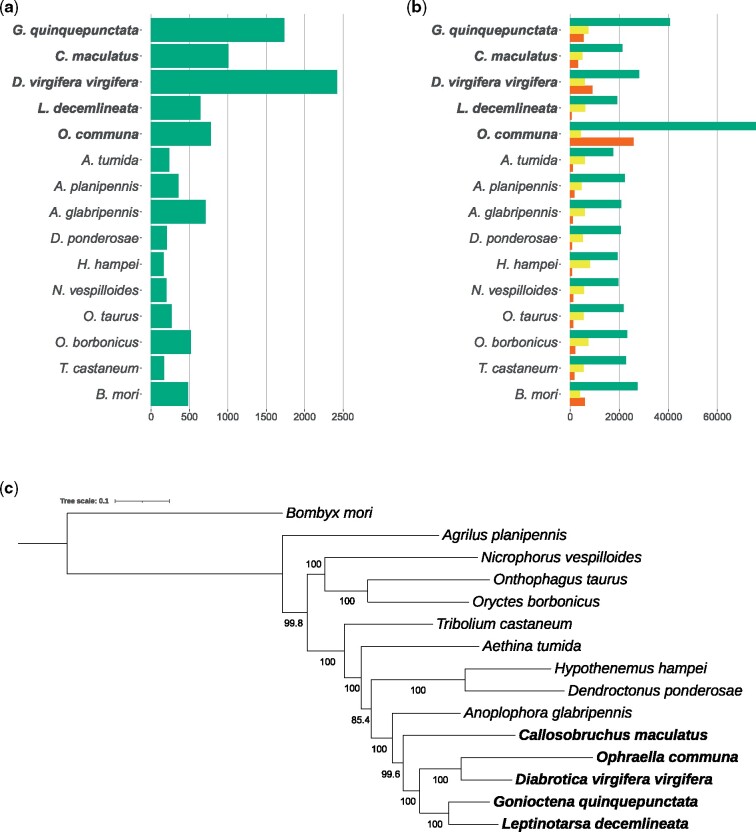
Comparison of the genome characteristics of *G. quinquepunctata* with those of four other species of chrysomelid beetles (in bold), of nine other beetle species and one outgroup (*Bombyx mori*). A maximum-likelihood phylogenetic tree was estimated for these species from an amino-acid alignment of the 52 single-copy proteins found in all 15 genomes. 1*a*: Assembly lengths, in Mb. 1*b*: Total number of predicted proteins (in green), number of predicted single-copy proteins (in yellow) and number of predicted species-specific proteins (in red). 1*c*: ML tree; bootstrap support values indicated along interior branches.

Orthofinder sorted 36,936 (91%) of the 40,568 proteins predicted for *G. quinquepunctata* into 12,978 orthogroups. This was the highest number of orthogroups identified of all species included in the analysis and represents 49.5% of the total number of orthogroups. In total, 1,471 (11.3%) of the orthogroups identified in the genome of *G. quinquepunctata* were species-specific and included 9,095 genes. Among all predicted genes, 7,367 (18.1%) were identified as single copy (i.e. present only once in their orthogroup). The phylogeny estimated (fig. 1) from the 52 single-copy genes found in every one of the 15 compared species is fully compatible with that of more comprehensive phylogenetic studies of the Coleoptera (e.g., McKenna et al. 2019).

## Materials and Methods

### Insect sampling, DNA and RNA Extraction, Sequencing

Sampling of *G. quinquepunctata* was conducted in the Vosges mountains (France), where its sister species *G. intermedia* is absent (P.M.’s unpublished observations), to avoid collecting hybrid individuals. DNA extraction was performed on a single pupa collected on 14 May 2018 in the vicinity of the “Col d’Urbeis” (48.330 N, 7.174 E), using the Qiagen kit Genomic-tip 20/G following manufacturer’s protocol.

About 1.5 µg of genomic DNA was sent to Genewiz (www.genewiz.com) for library preparation and DNA sequencing on an Illumina HiSeq 2500 platform, which resulted in 145.1 Gb of data (approximately 290 million pairs of PE reads 2 × 250 b). An additional 1.3 µg was used for Nanopore library preparation and sequencing. Five libraries were prepared using the SQK-LSK109 Nanopore kit. Sequencing was performed on a MinION sequencer with five flow cells version 9.4, generating 46.3 Gb of data (4.4 million reads with lengths ranging from 31 to 144,886 bp).

RNA was extracted from four individuals at different developmental stages (all collected on 19 June 2018), using the Qiagen RNeasy Mini kit following the manufacturer’s protocol: one adult male and one fourth-instar larva collected in the vicinity of “Grand Ballon” (47.90 N, 7.103 E), one pupa collected in the vicinity of “Le Breitfirst” (47.95 N, 7.023 E), as well as one adult collected in the vicinity of “Col d’Urbeis” (same coordinates as before). The RNA extracts were sent to Eurofins Genomics (www.eurofinsgenomics.eu) for library preparation and RNA sequencing on an Illumina HiSeq 2500 platform, which resulted in 51.2 Gb of data (a total of 177 million pairs of PE reads [2 × 150 pb]).

### Genome Assembly

Genome size and heterozygosity were estimated using GenomeScope (online version) v.2.0 (Vurture et al. 2017; Ranallo-Benavidez et al. 2020) and the kmercountexact tool of BBTools v.37.55 (https://sourceforge.net/projects/bbmap/) with a k-mer size of 31 for both programs. GenomeScope was run on a k-mer spectrum computed using Jellyfish v.2.3.0 (Marçais and Kingsford 2011) with the option -C to count canonical k-mers.

The genome of *G. quinquepunctata* was assembled using wtdbg2 v.2.5 (Ruan and Li 2020), a long-read assembler that does not require much resources (Guiglielmoni et al. 2021), with the following parameters: -x ont -g 1.5 g -t 16. A consensus was obtained using wtpoa-cns then polished using the same tool after aligning the Nanopore sequences on the contigs using minimap2 v.2.17 (Li 2018) and processing the output using SAMtools v.1.9 (Li et al. 2009; Li 2011). It was then polished once by running wtpoa-cns on the Illumina paired-end sequences aligned on the contigs using bwa v.0.7.17-r1188 (Li and Durbin 2009), following wtdbg2’s README.md file. Prior to the polishing step, the adapter sequences were trimmed from the Illumina reads using BBDuk of BBTools v.35.80 with the options minlen = 100 ktrim=r k = 25 mink = 11 hdist = 1 tpe tbo --ordered.

Duplicated regions were removed from the resulting assembly using Purge Haplotigs (Roach et al. 2018). The absence of cloning vector and synthetic sequences (adapters, linkers, and primers) in the curated contigs was checked by comparing them to the UniVec database (https://www.ncbi.nlm.nih.gov/tools/vecscreen/univec/) using BLAST as specified on the VecScreen page (https://ftp.ncbi.nlm.nih.gov/pub/UniVec/) and manually corrected. The resulting assembly was evaluated using QUAST v.5.0.2 (Gurevich et al. 2013) and BUSCO v.3.1.0 (Simão et al. 2015) using the database insecta_odb9 comprising 1,658 core genes. K-mer spectra plots and k-mer completeness were generated using KAT v.2.4.2 (Mapleson et al. 2017) on the Illumina sequences with default parameters.

### Genome Annotation and Phylogenetic Analysis

Prior to annotating the genome, a species-specific repeat library was built using RepeatModeler v.2.0.1 (Flynn et al. 2020) with the option -LTRStruct. This library, in combination with the Repbase library (RepeatMasker edition 20181026, Bao et al. 2015) was used to search and mask repeats in the genome using RepeatMasker v.4.1.1 (Smit et al. 2013–2015). RepeatMasker was run with the following options: -e ncbi -xsmall -poly -html -gff -source -frag 6000000.

The masked *G. quinquepunctata* reference assembly was then annotated with BRAKER2 v.2.1.5 (Stanke et al. 2008; Hoff et al. 2016, 2019; Brůna et al. 2021) using the RNA-Seq library as evidence. RNA-Seq data were filtered following the protocol described in Freedman and Weeks (2020) and mapped to the *G. quinquepunctata* reference assembly using HISAT2 v.2.1.0 (Kim et al. 2015, 2019). The resulting SAM file was sorted using SAMtools v.1.9. BRAKER2 was run with the --bam, --softmasking, and --gff3 parameters, using DIAMOND v.2.0.7.145 (Buchfink et al. 2015), SAMtools v.1.9 and Augustus v.3.3.3 (Stanke et al. 2006).

The genes predicted were annotated by comparing them to the Swiss-Prot and NR databases (downloaded in March 2021) using BLASTP v.2.9.0+ (Altschul et al. 1990; Camacho et al. 2009) and selecting the best hits with *e*-values below $10^{-5}$. A second annotation was performed using InterProScan v.5.50-84.0 (Jones et al. 2014) with default parameters. The InterProScan results were then filtered to remove all matches with *e*-value greater than $10^{-5}$ and the match with the lowest *e*-value was kept for each gene.

A phylogenetic analysis to search for orthologous genes was conducted using OrthoFinder v.2.5.2 (Emms and Kelly 2015, 2019), comparing the predicted genes found in *G. quinquepunctata* to those of four other species of chrysomelid beetles: *C. maculatus* (Sayadi et al. 2019), *Diabrotica virgifera virgifera* (NCBI, BioProject: PRJNA432972), *L. decemlineata* (Cunningham et al. 2015), and *O. communa* (Bouchemousse et al. 2020); of nine other beetle species: *Aethina tumida* (Evans et al. 2018), *Agrilus planipennis* (NCBI, BioProject: PRJNA230921), *A. glabripennis* (McKenna et al. 2016), *Dendroctonus ponderosae* (Keeling et al. 2013), *Hypothenemus hampei* (Vega et al. 2015), *Nicrophorus vespilloides* (Cunningham et al. 2015), *Onthophagus taurus* (NCBI, BioProject: PRJNA167478), *Oryctes borbonicus* (Meyer et al. 2016), and *Tribolium castaneum* (Herndon et al. 2020); and of *Bombyx mori* (Kawamoto et al. 2019) as an outgroup (fig. 1 and supplementary table 1, Supplementary Material online). Once genes were sorted in orthogroups and single-copy genes were identified, we inferred a species tree from all 52 single-copy genes that were present in every 15 species. Alignments of protein sequences were conducted using MUSCLE v.3.8.31 (Edgar 2004) then concatenated into a single data set using FASconCAT-G v.1.04 (Kück and Meusemann 2010). The best-fit partitioning scheme and the best model for each partition were selected using ModelFinder (Kalyaanamoorthy et al. 2017) with options -m TESTMERGEONLY -mset mrbayes -rcluster 10, then a maximum-likelihood tree search was performed using IQ-TREE v.2.0.6 (Nguyen et al. 2015) with ultrafast bootstrapping (Hoang et al. 2018) and with Shimodaira–Hasegawa approximate likelihood ratio tests.

The possibility that this genome may have undergone a whole-genome duplication was tested using MCScanX (Wang et al. 2012) downloaded from https://github.com/wyp1125/MCScanX on March 27, 2021.

## Supplementary Material

Supplementary data are available at *Genome Biology and Evolution* online.

## Supplementary Material

evab134_Supplementary_Data
