# Supplementary material for: Genome Assembly of the Cold-Tolerant Leaf Beetle Gonioctena quinquepunctata, an Important Resource for Studying Its Evolution and Reproductive Barriers between Species
Source: Genome Biol Evol. 2021 Jun 11;13(7):evab134. doi: 10.1093/gbe/evab134 (PMC8290105; doi:10.1093/gbe/evab134)
Supplement: evab134_Supplementary_Data [file evab134_supplementary_data.zip › Supplementary material.pdf]

(a)

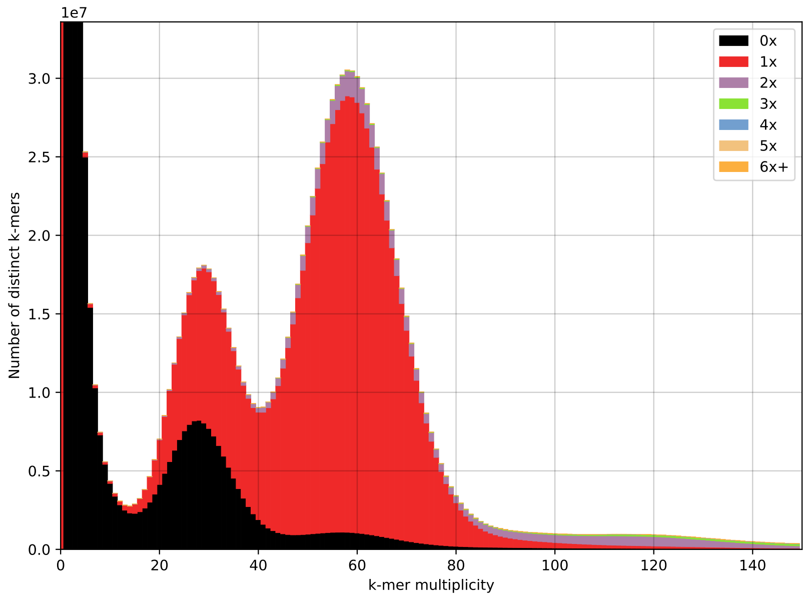

(b)

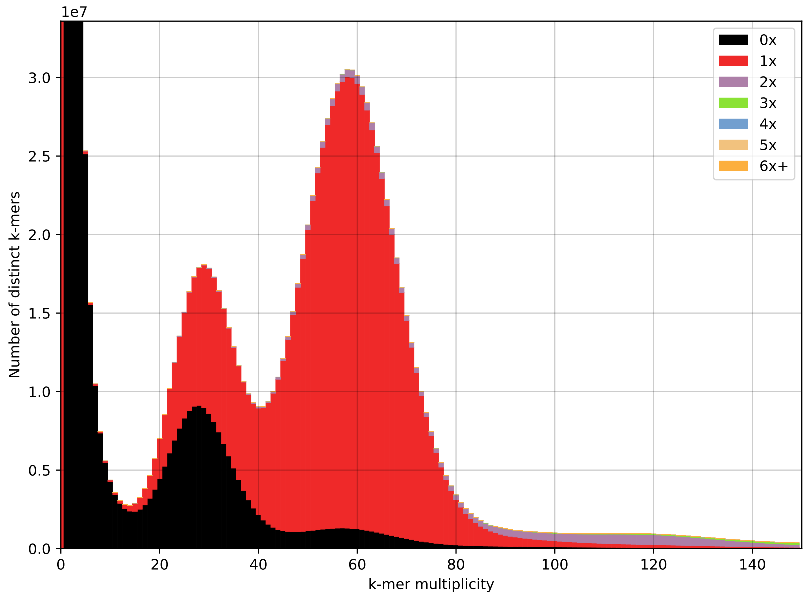

Fig. 1: Comparison of k-mer spectra generated using KAT before (1a) and after (1b) running Purge Haplotigs. Running Purge Haplotigs decreased the number of k-mers represented twice in the assembly.

Table 1. Species used for the phylogenetic analysis.

Type	Species	Common name	Sequences	Assembly size (Mb)	Scaffold N50 (kb)	Accession number
Chrysomelidae	*Callosobruchus maculatus*	Cowpea weevil	31,657	1,007	212	GCA_900659725.1
	Diabrotica virgifera virgifera	Western corn rootworm	28,061	2,418	489	GCA_003013835.2
	Gonioctena quinquepunctata		40,568	1,732	432	Present study
	Leptinotarsa decemlineata	Colorado potato beetle	19,038	641	139	GCA_000500325.2
	Ophraella communa	Ragweed leaf beetle	75,642	774	195	GCA_902651945
Coleoptera	*Aethina tumida*	Small hive beetle	17,463	234	298	GCA_001937115.1
	Agrilus planipennis	Ash borer	22,159	353	1,113	GCA_000699045.2
	Anoplophora glabripennis	Asian longhorned beetle	20,632	710	659	GCA_000390285.2
	Dendroctonus ponderosae	Mountain pine beetle	20,551	204	581	GCA_000355655.1
	Hypothenemus hampei	Coffee berry borer	19,222	163	44	https://genome.med.nyu.edu/coffee-beetle/cbb.html
	Nicrophorus vespilloides	Burying beetle	19,577	195	122	GCA_001412225.1
	Onthophagus taurus	Taurus scarab	21,668	267	337	GCA_000648695.2
	Oryctes borbonicus	Scarab beetle	23,278	518	104	LJIG00000000.1
	Tribolium castaneum	Red flour beetle	22,610	165	4,753	GCF_000002335.3
Outgroup	*Bombyx mori*	Domestic silkworm	27,309	481	4,008	GCA_014905235.2
